# Prevalence of children born small for gestational age with short stature who qualify for growth hormone treatment

**DOI:** 10.1186/s13052-021-01026-3

**Published:** 2021-04-01

**Authors:** Gianluca Tamaro, Mariagrazia Pizzul, Giuliana Gaeta, Raffaella Servello, Marina Trevisan, Patricia Böhm, Paola Manera Ada Materassi, Anna Macaluso, Denis Valentini, Maria Chiara Pellegrin, Egidio Barbi, Gianluca Tornese

**Affiliations:** 1 University of Trieste, Trieste, Italy; 2 Primary Care Pediatrician, Trieste, Italy; 3 Institute for Maternal and Child Health IRCCS “Burlo Garofolo”, Via dell’Istria 65/1, 34137 Trieste, Italy

**Keywords:** Small-for-gestational age, Growth hormone, Short stature, Catch-up growth, Italy

## Abstract

**Background:**

Recombinant human growth hormone (rhGH) is approved in Europe as a treatment for short children born small for gestational age (SGA) since 2003. However, no study evaluated the prevalence of SGA children with short stature who qualify for rhGH in Europe so far. This study aimed to investigate in an Italian population the prevalence of children born SGA, of short stature in children born SGA, and of SGA children who qualify for rhGH treatment at 4 years of age.

**Methods:**

We conducted a population-based study on primary care pediatricians’ databases in Trieste, Italy. Data was collected on 3769 children born between 2004 and 2014. SGA was defined as birth weight and/or birth length ≤ − 2 SDS. Data on height and weight were registered at the closest well-being visit to 1, 2, 3, 4 years of age. Short stature was defined as height ≤ − 2 SDS. Short children born SGA who qualify for rhGH treatment were identified according to Note AIFA #39 criteria (age ≥ 4 years; height ≤ − 2.5 SDS; growth velocity < 50th percentile).

**Results:**

Full data at birth were available for 3250 children. The SGA prevalence was 3.6% (0.8% SGA for weight, 2.2% SGA for length, 0.6% SGA for both weight and length). The prevalence of short stature among SGA children was 9% at 1 year of age, 6% at 2 years (significantly higher in preterm in the first 2 years), 4% at 3 years, 3% at 4 years (all born at term). At 4 years of age, median height SDS was − 0.52. One child born SGA was eligible for GH treatment (0.8% among SGA children).

**Conclusions:**

The prevalence in a general pediatric population of children born SGA who qualify for GH treatment was 1:3250. Although the prevalence of SGA in our population was similar to previous studies, catch-up growth was recorded earlier in our sample compared to previous reports, and term babies had late catch-up. Height SDS of children born SGA at 4 years of age was lower than expected (− 0.52 SDS).

## Background

Children born small for gestational age (SGA) – defined when birth weight (BW) or birth length is ≤ − 2 standard deviations score (SDS) for gestational age (GA) [1] – represent 3.1–5.5% of the population [2–4]. The causes of SGA are multifactorial and include maternal lifestyle and obstetric factors, placental dysfunction, and fetal (epi)genetic abnormalities. These children may present several growth, hormonal and developmental peculiarities, possibly due to the growth restriction developed during pregnancy, which may lead to health consequences in later life; for this reason, a long-term multidisciplinary follow-up should be warranted in order to monitor and improve the long-term outcomes [5, 6].

Persistent short stature is one of the most frequent complications of being born SGA: these children often present a catch-up growth that is more pronounced during the first 6 months and is usually completed in the first 2 years of life. Nevertheless, previous studies found that approximately 10% of these children do not catch up by 2–3 years of age [1, 7], and they will have a higher risk of short stature later in life [8–10]. A population-based study conducted in Sweden indicated that SGA subjects who did not reach early childhood growth constitute 21% of short prepubertal children and 8% of short individuals at 18 years of age [11].

Recombinant human growth hormone (rhGH) is an approved and effective treatment for short children born small for gestational age (SGA) [1, 12]. This treatment was approved in 2001 from the American Food and Drug Administration (FDA), in 2003 from the European Medicines Agency (EMA) and in 2008 from the Ministry of Labor and of Welfare in Japan; eligibility criteria are slightly different among several countries: for instance, rhGH can be prescribed at the age of 2 years in the USA, 3 years in Japan and 4 years in Europe [1].

Italian Medicines Agency (AIFA) approved rhGH to treat children with short stature born SGA in 2009. The drug is reimbursed by the Italian National Health System (Servizio Sanitario Nazionale – SSN) according to Note #39 on the use of drugs. Since 2014, to access treatment with rhGH in individuals born SGA in Italy, it is necessary to meet all the following criteria (in line with EMA indications) [13]:

- BW and/or BL ≤ − 2 standard deviations score (SDS) for gestational age (GA) according to Bertino charts [14];
- age at the start of GH therapy ≥4 years;
- height ≤ − 2.5 SDS;
- growth velocity < 50th percentile.

The hypothetical prevalence of short children born SGA at the age of 2 years would be 0.24% (12% of 2%, 1:417) [15]; however, no study evaluated the prevalence of SGA children with short stature who qualify for rhGH treatment in Europe, so far. Only one Japanese study on a cohort of nearly 30,000 children re-evaluated at 3 years of life verified the prevalence of children with short stature born SGA of 0.06% (1:1800) (notably higher in preterm births < 34 weeks of GA, 0.39%, 1:256) [2].

This study aimed to investigate in an Italian population the prevalence of:

- children born SGA;
- short stature in children born SGA at the age of 1, 2, 3, and 4 years;
- SGA children who qualify for rhGH treatment at 4 years of age.

## Materials and methods

We conducted a population-based study on children born between 2004 and 2014 (when the present version of Note #39 was approved) and who had at least 4 years of follow-up in 2018. Anonymized data were retrieved by 7 out of 20 primary care pediatricians’ digital databases in the province of Trieste, Italy. The remaining 13 primary care pediatricians could not be involved because they retired during the study period or did not have access to electronic archives. Overall, data on 3769 children (over 20,120 [16]) were collected.

Information from the birth, such as BW, BL, and GA, were recorded by primary care pediatricians directly from the neonatal hospital reports into the database during the first visit. Data on height were collected during well-child visits, and those closest to 1, 2, 3, 4 years of age were retrieved.

BW and BL SDS for sex, GA, and birth order were calculated according to Bertino Italian charts [14] through the website (http://www.inescharts.com) designed by scientific societies (Italian Society of Pediatric Endocrinology and Diabetology – SIEDP, Italian Society of Neonatology – SIN, Italian Society of Medical Statistics and Clinical Epidemiology – SISMEC). SGA was defined as BW and/or BL ≤ − 2 SDS.

Height SDS was determined through Growth Calculator distributed by SIEDP on the website http://www.weboriented.it/gh4/. Short stature was defined as height ≤ − 2 SDS according to the World Health Organization (WHO) charts for children < 5 years [17].

Short children born SGA who qualify for rhGH treatment were identified as meeting all of the above criteria.

Ethical Committee approval was not requested since General Authorization to Process Personal Data for Scientific Research Purposes (Authorization no. 9/2014) declared that retrospective archive studies that use ID codes, preventing the data from being traced back directly to the data subject, do not need ethics approval [18].

Statistical analyses were mainly descriptive. Data are presented as frequencies and percentages or as median and interquartile ranges (IQRs). Mann-Whitney rank-sum tests and Two-tailed Fisher exact tests were performed to evaluate the relations between variables. Wilcoxon signed-rank test was used to check the differences of paired data. Analyses were conducted using JMP*™* software (version 15.1.0, SAS Institute Inc., Cary, NC, United States).

## Results

Full data at birth were available for 3250 children; 519 children were excluded because of incomplete data.

Overall, 118 children (53 females) were born SGA (3.6%): 26 for weight (SGA-W, 0.8%), 72 for length (SGA-L, 2.2%) and 20 for both weight and length (SGA-WL, 0.6%) (Table 1). All children born SGA-W (including SGA-WL) were significantly leaner than SGA-L (*p* < 0.01). All children born SGA-WL were significantly shorter than SGA-L, and both were significantly shorter than SGA-W (*p* < 0.01). Males were more represented in SGA-W than in SGA-L (*p* = 0.03). Thirteen children (11%) were preterm (< 37 weeks of GA), and 104 (89%) were term (≥37 weeks of GA) babies.

**Table 1:** Descriptive data on children born small for gestational age (SGA) for weight (SGA-W), for length (SGA-L), or weight and length (SGA-WL). Data are reported as frequencies, percentages, and medians (interquartile ranges) (° *p* = 0.03; */§/# *p* < 0.01 considered between SGA classes)

	n	% on all newborns	Female n (%)	Preterm n (%)	Weight kg	Weight SDS	Lenght cm	Lenght SDS
*SGA-W*	26	0.8%	7 (28%)°	5 (20%)	2.40 (2.15;2.57)*	−2.2 (−2.5;-2.1)*	47 (46;48)*#	−1.7 (−1.8;-1.1) *#
*SGA-L*	72	2.2%	38 (52%)°	18 (25%)	2.76 (2.46;3.03)*§	−1.3 (−1.8;-0.8)*§	45 (43;46)*	−2.2 (−2.6;-2.1) *§
*SGA-WL*	20	0.6%	8 (40%)	3 (15%)	2.39 (2.22;2.54)§	−2.2 (− 2.3;-2.1)§	45 (42;46)#	− 2.6 (−3.2;-2.4) §#
*All SGA*	118	3.6%	53 (45%)	26 (22%)	2.57 (2.36;2.90)	−1.8 (−2.2;-1.06)	45 (43;47)	−2.3 (− 2.6;-2.1)

**Fig. 1:**
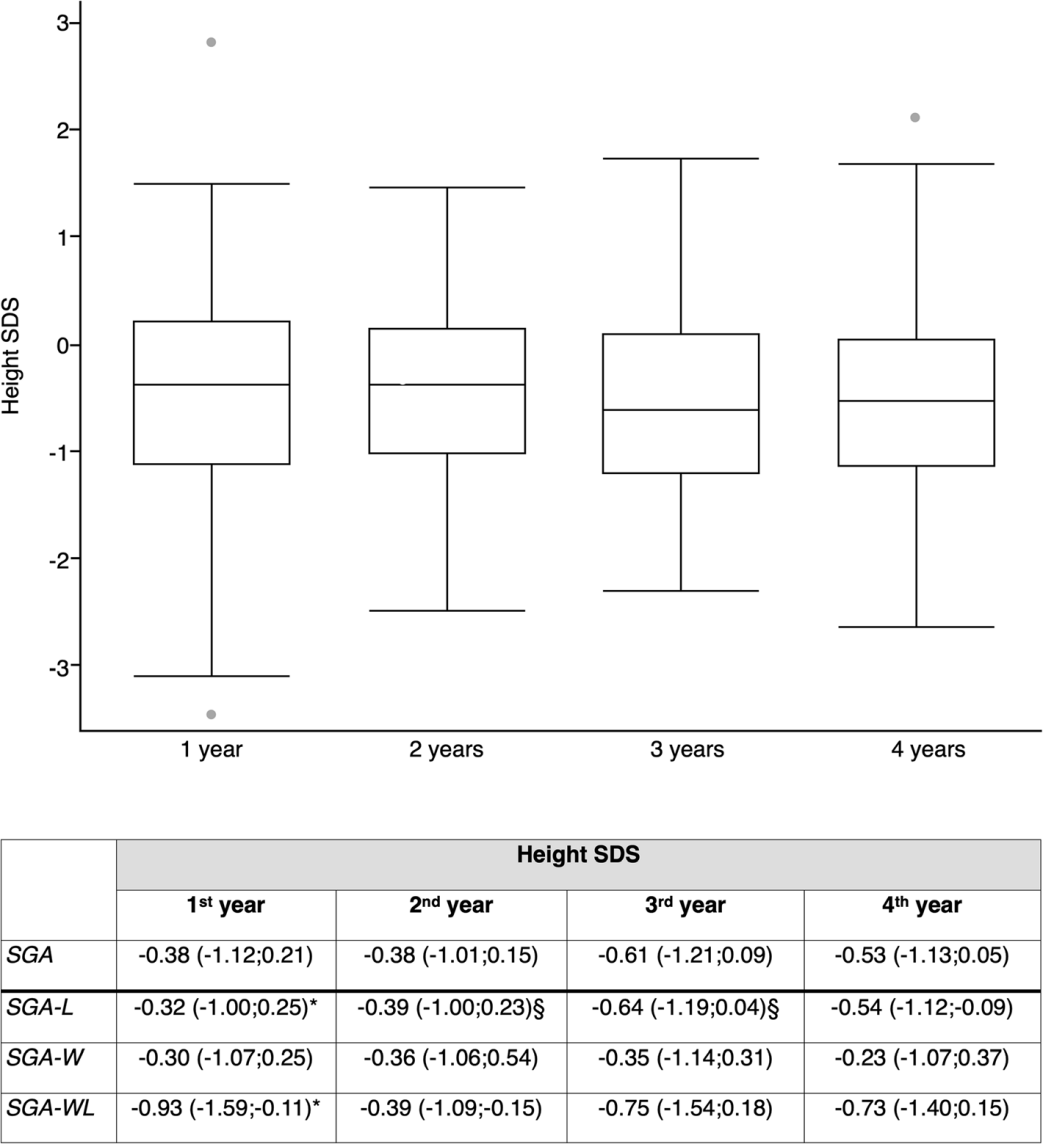
Height SDS over 4-year follow-up (data are reported as medians and interquartile ranges) (*differences between SGA-WL and SGA-L/SGA-W, *p* = 0.03)

Data on height SDS at 1, 2, 3, and 4 years are reported in Fig. 1. At 1 year of age, SGA-WL were significantly shorter (− 0.93 SDS) than others (*p* = 0.03), while no differences were found in the following years. At the age of 4, median height SDS in SGA children was − 0.53; children born SGA-WL were shorter (− 0.73 SDS) than those born SGA-L (− 0.54 SDS) and SGA-W (− 0.23 SDS), although these differences did not reach statistical significance.

**Fig. 2:**
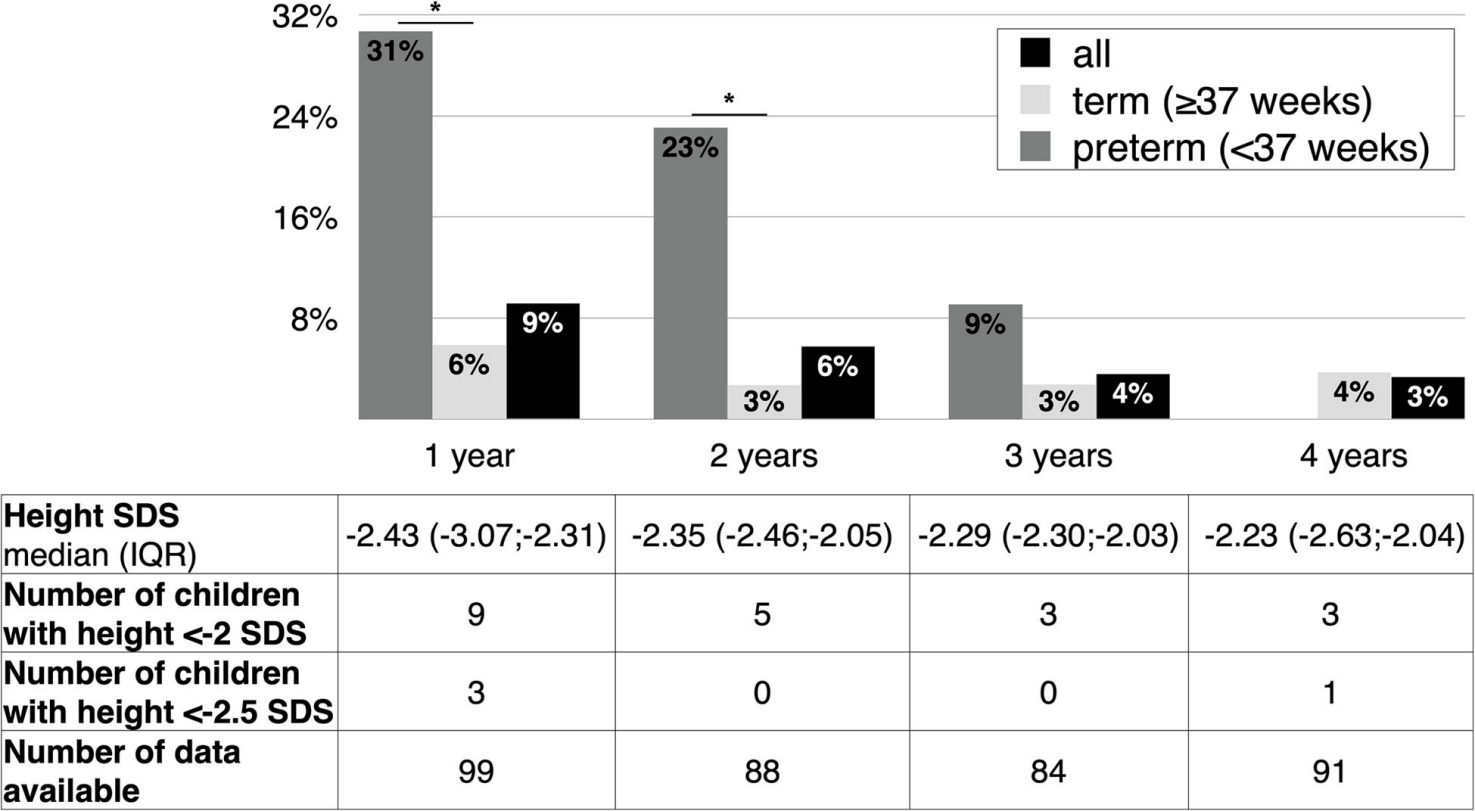
Short stature in children born SGA. The graph reports the rate of children <− 2 SDS at 1, 2, 3, and 4 years of age by gestational age group (preterm: < 37 weeks; term: ≥37 weeks) and for all the sample; short stature rates at each age were compared between gestation groups (*p < 0.01). The table reports height SDS (median and IQR) and the number of children with height < − 2 SDS, <− 2.5 SDS, and those for which data about height were available over the years

Short stature rate of children born SGA was 9% at 1 year, 6% at 2 years, 4% at 3 years, and 3% at 4 years; SGA children born preterm had a significantly higher prevalence of short stature at 1 and 2 years of age compared to those born at term (*p* < 0.01) (Fig. 2). No differences were found in sex or birth order.

At 4 years of age, three children (all born at term) were short in stature, with a growh velocity < 50th percentile, but only one (0.8% among SGA children) presented a stature <− 2.5 SDS, meeting all the criteria for rhGH prescription. If we had considered children with no evidence of catch-up at the age of 2 or 3 years, no children would have been treated because heights were > − 2.5 SDS (Fig. 2).

## Discussion

In this retrospective study, we analyzed data on 3250 children over 20,120 born between 2004 and 2014 in the province of Trieste, with a 4-year auxological follow-up by primary care pediatricians. To our knowledge, this is the first study in Italy that comprehensively considered the prevalence of short stature in children born SGA and the first in Europe, which considered SGA children who qualify for rhGH treatment at 4 years of age.

The SGA prevalence in our study (3.6%) was similar in previous studies in Finland (3.1%) [3] and Japan (3.5%) [2], lower than that reported in Sweden (5.5%) [8] and in a previous Italian study in the Chieti province (14.5%) in which – however – a different definition of SGA was used (<− 1.28 SDS) [19].

Catch-up growth was recorded earlier in our sample compared to previous reports: at the age of 1 year, 91% of children born SGA had a height > − 2 SDS (vs. 68% in Japan [7] and 87% in Sweden [11]), at the age of 2 the rate was 94% (vs. 89% in Japan and 87% in Sweden), at the age of 4 the rate was 97% (vs. 88% in Japan and 92% in Sweden at the age of 5); term babies had catch-up growth earlier than pre-term, as reported in previous studies [20–22]. These data seem to justify the European indication for waiting up to the age of 4 years, since there is a slight possibility of spontaneous catch-up growth between 2 and 4 years of age, not only in children born prematurely, as previously reported [23, 24], but – according to our data – also in those born at term. While it is well known that SGA infants differ in postnatal growth, the complex mechanism underlying is difficult to unveil. Different anomalies in the GH-IGF1 axis had been described [25, 26], although circulating concentrations of GH, IGF-I, IGF-BP3 were found not to be predictive of subsequent growth [27]. MicroRNAs have been described as novel biomarkers for the early identification of catch-up growth in SGA infants, in particular miR-576-5p – with whom insulin, IGF-1, PDGFR-B, and mTOR signaling pathways are associated – seems to contribute to the regulation of postnatal growth and potentially influence the risk for cardiometabolic diseases associated in SGA children [28]. An ultrasound parameter, the evaluation of neonatal bone maturation by studying Béclard’s nucleus - has been recently found to be a predictive factor of SGA height gain during the first year of life [29].

Remarkably, the median height of children born SGA at 4 years of age in our cohort was lower than the general population (− 0.52 SDS), and children born SGA-WL had a median height (− 0.73 SDS) that was lower compared to SGA-L and SGA-W (− 0.54 and − 0.23 SDS, respectively), as previously reported [4], although this difference did not reach statistical significance.

The prevalence in the general pediatric population of children born SGA who qualify for rhGH treatment at 4 years of age in our cohort was 1:3250, smaller than the hypothetical one (1:417) [17] and than previously reported (1:1800 in Japan at 3 years of age) [2], but still very far from the stated prevalence of children treated with rhGH with SGA indication in Italy (0.37/100,000) [15]. Intriguingly, all children with short stature at the age of 4 years were born at term, and if we had considered the age of 2 years (as in the USA) or 3 years (as in Japan), no children would have qualified for rhGH treatment, considering all the remaining criteria. A prolonged follow-up in children born SGA is crucial throughout all infancy, even after the age of 2 years and in term babies. Recent studies confirmed that monogenic conditions (mostly related to the growth plate) play an important role in short stature, especially among SGA children [30, 31]. For instance, mutations in the *IHH* gene are associated in children born SGA with short stature (with nonspecific skeletal abnormalities) that may appear later in infancy; these children seem to have a good response to the rhGH treatment [30]. Genetics studies will probably answer the question related to the highly variable response to rhGH therapy in SGA children [32], and other short stature conditions.

This study has some limitations, mainly due to its retrospective design. Anthropometric parameters were assessed by different physicians and not standardized; 13/20 primary care pediatricians could not be involved, leading to an analyzed sample which is not completely representative of the entire population of Trieste province; since data were anonymized, we could not retrieve any information about concomitant diseases that can interfere with growth (such as suspected or recognized genetic syndromes) or other additional data (such as ethnicity of the enrolled children).

Nevertheless, this study has a sample size comparable to that of previous studies on this topic, reflects real-life clinical practice and provides data that were not reported before and useful in clinical practice (follow-up in SGA children) and in guiding public health interventions (prevalence of children who qualify for the rhGH treatment according to SGA indication).

## Conclusions

In conclusion, while the prevalence of SGA children in our population is similar to previous studies, data on catch-up growth are different from previous reports, with earlier catch-up growth. Prolonged follow-up – at least until the age of 4 years, but probably more extended – is needed not to lose children that fail to catch-up (even among term babies) and may require rhGH treatment (even though their prevalence is smaller than previously reported), but also to monitor and improve the long-term outcomes.

## Data Availability

The data that support the findings of this study are available from the corresponding author, GTo, upon reasonable request.

## Abbreviations

AIFA: Italian Medicines Agency
BL: Birth length
BW: Birth weight
EMA: European Medicines Agency
FDA: Food and Drug Administration
GA: Gestational age
GHD: Growth hormone deficiency
IQR: Interquartile range
rhGH: Recombinant human growth hormone
SDS: Standard deviation score
SIEDP: Italian Society of Pediatric Endocrinology and Diabetology
SIN: Italian Society of Neonatology
SISMEC: Italian Society of Medical Statistics and Clinical Epidemiology
SGA: Small for gestational age
SGA-L: Small for gestational age for length
SGA-W: Small for gestational age for weight
SGA-WL: Small for gestational age for weight and length
SSN: Italian National Health System
WHO: World Health Organization
